# Falls prevention for older adults in outdoor public spaces: an interdisciplinary Delphi consensus on risks, actions, and barriers

**DOI:** 10.1186/s12889-026-27548-1

**Published:** 2026-05-02

**Authors:** Antoine Langeard, Marion Torterotot, Marine Le Roux, Bettina Wollesen

**Affiliations:** 1 Inserm, CYCERON, COMETE U1075, Université de Caen Normandie, Caen, France; 2 Cerema, Direction territoriale Normandie-Centre, Le Grand-Quevilly, France; 3 Gérontopôle Normandie, Caen, France; 4 Institute of Movement Therapy and Movement-Oriented Prevention and Rehabilitation, German Sports University Cologne, Cologne, Germany

**Keywords:** Older adults, Falls in outdoor public spaces, Delphi, Prevention, Urban planning

## Abstract

**Background:**

Falls are the leading cause of accidental injury among older adults, 30% of community-dwelling adults aged 65 and over fall each year, with nearly half occurring outdoors. These falls are complex, understudied, and insufficiently addressed in current age-friendly cities or walkability frameworks. This study aimed to build interdisciplinary consensus on risks, preventive actions, and barriers to fall prevention in outdoor public spaces through a Delphi process.

**Methods:**

A three-phase Delphi study was conducted with 64 participants in round 1, 60 in round 2, and 49 in round 3, including four expert groups: older adults who had fallen outdoors, health and research professionals, urban planners, and decision-makers (local and regional policy-makers, elected officials, and public-space managers involved in urban planning). Phase one collected open responses on risks, preventive actions (modification of physical layout, public-space management, and behavior-related factors), and barriers to these actions. Responses were synthesized using AI-assisted analysis with systematic human validation. In phases two and three, the relevance of 124 propositions were rated on a 10-point Likert scale. Consensus was defined as ≥ 70% of ratings ≥ 7/10 and interquartile range ≤ 2.5.

**Results:**

Consensus was reached for key intrinsic factors such as gait and balance impairments, visual and vestibular deficits, cognitive decline, and polypharmacy, as well as for environmental factors including irregular or inappropriate surfaces, obstacles, or signage, and crowding. Highly relevant preventive actions included integrating fall prevention into street and sidewalk design, training urban planning professionals, awareness campaigns, systematic maintenance, safer crossings, participatory co-design public-space adaptations and urban design features involving older adults and local stakeholders, and improved data monitoring through surveillance, mapping, and sharing of fall-related and environmental risk information. Main barriers were insufficient budgets, high costs, limited integration of fall prevention into planning priorities, and lack of evaluation of the impact of implemented actions.

**Conclusions:**

Outdoor fall prevention is a transversal challenge requiring integration of public health and urban planning. This Delphi highlights actionable priorities to embed fall prevention in local and national strategies, in particular in rapidly aging regions.

**Supplementary Information:**

The online version contains supplementary material available at 10.1186/s12889-026-27548-1.

## Introduction

Falls are the leading cause of accidents in daily life among older adults. The consequences are severe, as falls can result in fractures, loss of independence, hospitalizations, and even death. Beyond the physical consequences, falls also have a profound psychological impact, as they can lead to a loss of confidence in one’s balance, resulting in social isolation and progressive withdrawal [1, 2]. With approximately 30% of adults over 65 falling each year and associated healthcare costs exceeding 50 billion dollars in the United States alone [3], falls represent a major public health challenge, for which the overall impact of current strategies remains limited, as age-standardized fall incidence rates have not shown a consistent decline in Western Europe [4, 5]. By 2030, one in six people will be over 60 years old, and this population will have doubled by 2050 [6] making this issue even more pressing.

Available studies suggest that a substantial proportion of falls among community-dwelling older adults occur outdoors, particularly among more active individuals, with about 75% of outdoor falls triggered by environmental factors [7, 8], highlighting the crucial role of urban design. For older adults, moving around safely in public space is essential to maintaining social life, autonomy, an active lifestyle, preventing other health risks, accessing healthcare, shops, and public services [9]. Even though these falls present specificities (involving more active older adults and triggering more severe injuries [10, 11]), they remain largely understudied compared to falls indoors [7]. This is partly due to outdoor environments being more variable, shared among multiple users, and dependent on a wide range of stakeholders: urban planners, local authorities, technical services, equipment designers, health professionals, and citizens themselves. Outdoor falls thus represent a “wicked problem,” at the intersection of multiple domains (health, urban planning, mobility, social policy), with no single stakeholder being exclusively responsible [12]. International tools and frameworks relating to age-friendly urban environments (such as the AFCCQ questionnaire or the WHO Age-Friendly Cities framework) address accessibility, participation, and mobility broadly, but integrate little to no focus on fall prevention [13, 14]. In summary, outdoor falls can still be considered a neglected public health problem [7].

Very few scientific studies have so far systematically documented the risk factors for falls among older adults in outdoor public spaces, and they have simply relied on fallers’ recollections to identify types of fall risks [8, 10, 15]. This approach can be affected by recall bias and does not provide actionable solutions for fall prevention. Faced with this lack of consolidated data, the Delphi method appears particularly relevant. It is an iterative consensus-building approach among experts, conducted through several anonymous rounds where responses are aggregated, shared, and re-evaluated. This method is especially suited to contexts where knowledge is fragmented but where experiential knowledge, professional expertise, and field observations are diverse and complementary [16–19]. This is the case for fall risks in outdoor public spaces, making such type of study valuable in generating shared knowledge and priorities from heterogeneous opinions (urban planners, health professionals, local authority representatives, researchers, older adults who have fallen). Moreover, using the Delphi method fits fully within a broader participatory approach: integrating stakeholders and engaging users as co-producers of knowledge, strengthens the legitimacy, relevance, and operational impact of the results.

The challenge of preventing falls in outdoor public spaces is both local and global. Normandy, France, is a particularly critical setting, as it is experiencing one of the fastest growth rates of older adults in France [20]. This makes the region a prototype for demographic transition, where the interplay between rapid aging and diverse territorial structures can be studied in depth. Its urban fabric, comprising medium-sized towns, peri-urban areas, and historic centers, closely mirrors the traditional territorial configurations of many European regions. As such, studying falls in Normandy not only generates knowledge directly applicable to local public policies and stakeholder mobilization, but also provides transferrable insights for other regions navigating similar challenges of aging populations and urban adaptation.

In this context, the objective of the present study is to document, using an interdisciplinary classic Delphi method, the intrinsic (person-related) and extrinsic (environment-related) risk factors for outdoor falls among older adults, as well as the preventive actions and barriers to their implementation in public spaces. In this study, “outdoor public spaces” refer specifically to urban and community outdoor environments related to everyday mobility (e.g. sidewalks, roads, pedestrian crossings, public squares, bus stops, and parks), and exclude private spaces, buildings, transport vehicles, and nature-based recreational activities. This approach combines the expertise of urban planners, research and health professionals, local decision makers, and older adults who have themselves fallen in the outside environment, in order to identify a common core set of operational propositions. By bringing together a multidisciplinary stakeholder panel on this still under-researched topic, the study aims to fill a gap in the literature and provide concrete guidance for integrating fall prevention into urban planning and public health policies.

## Methodology

The Delphi method is a qualitative and prospective research method designed to find consensus among experts around a complex subject [16]. It is used in many fields when knowledge is still partial, fragmented, or dispersed among different stakeholders [16]. It relies on iterative, anonymous consultation of a panel of experts chosen for the complementarity of their experience and knowledge [19]. Contributions are then analyzed, synthesized, and redistributed to participants in successive predetermined cycles (usually three), enabling them to revise their views in light of the collective’s arguments [19]. The classic Delphi method used here is described below and followed current recommendations for health and social sciences [16–19, 21, 22]. The number of rounds was defined in advance to be a maximum of three rounds. The study was designed and reported in accordance with the DELPHISTAR guideline [18]. The checklist associated with the reporting guidelines for Delphi studies in social and health sciences (DELPHISTAR) is provided as Supplementary Material 1 [18]. Participation was anonymous and voluntary. All questionnaires were integrated into an online LimeSurvey. The University of Caen Normandy Research Ethics Committee approved the study protocol (no. 2025030408142400000260000327).

### Participants

This study was conducted in the context of the regional Anti-Fall Plan [23], a public health initiative coordinated at the regional level, within which voluntary thematic working groups were created to address specific fall-prevention issues. These groups are facilitated by designated coordinators whose role is to connect stakeholders from different sectors and support the emergence of collective actions, rather than to conduct research activities. The present Delphi study emerged from discussions within the working group dedicated to outdoor public spaces. The Delphi process relied on a panel of experts concerned in different ways with falls in outdoor public spaces. In order to restrain some homogeneity, the panelists were all from the Normandy region. The working group (WG) responsible for this study was composed of various stakeholders with experiential or professional knowledge of how outdoor public spaces could contribute to fall in older adults: older users, urban‑planning professionals, physicians and researchers on aging and falls, local decision‑makers, and associations. The working group was advised by external methodological experts with experience in Delphi studies. They decided to target four categories: (1) older adults aged 65 + who had already fallen outdoors, providing experiential expertise; (2) scientific or technical experts and health professionals, with clinical or research expertise (geriatricians, emergency physicians, GPs, researchers in falls prevention.); (3) planning experts, with professional expertise in the design and management of public space (technical municipal services, urban planners, architects, etc.); and (4) decision‑makers with governance expertise (local authority representatives, elected officials). Participants were considered experts by experience or by profession and were not constrained by a predefined operational definition of a fall. Names and emails were proposed by WG members and snowball recruitment was also used as recommended to improve Delphi sampling [22]. A total of 171 contacts were invited even if they had not responded previously. The target was to collect at least 60 responses in phase 1 and not drop below 47 in the final phase with at least 10 participants per expert group [19].

An invitation letter was emailed in early March, 2025 detailing objectives, confidentiality, schedule, and the survey link. Round 1 remained open for three weeks, with a reminder one week before closing.

The Delphi process comprised three rounds. Round 1 involved an open-ended questionnaire co-designed by the multidisciplinary working group and completed by all recruited panelists across the four expert categories. Rounds 2 and 3 were based on the structured synthesis of Round-1 responses and involved quantitative rating by the full panel, with iterative feedback provided between rounds to progressively refine items and reach consensus.

### Round 1

The first-round questionnaire was co‑designed within a regional “Anti‑Fall Plan” by the “Outdoor Public Space” WG composed of various stakeholders linked to older adults’ fall risk outdoors: older users, urban‑planning professionals, physicians and researchers on aging and falls, local decision‑makers, and associations. The objective of this first questionnaire was to collect information on risk factors in outdoor public spaces, on possible solutions to reduce these falls, and on potential barriers to these solutions. The WG’s initial meeting was held in March 2024, during which the method was presented, and the working group decided to organize thirteen open questions into three sections: causes of falls; actions to adapt public space; and barriers and facilitators to implementing prevention actions. Each question proposed by the members were then discussed and revised based on others’ feedback. Causes of falls were explored via five open questions related to intrinsic factors (person‑related), extrinsic factors (environment‑related) that could decrease or increase fall risk. Actions to prevent falls were explored by four open questions targeting modifications to physical layout, space management, and behaviors. Barriers and facilitators were explored by two open questions on difficulties in implementing proposed actions and on enablers. Two final open questions captured any information not covered earlier. The questionnaire is provided as Supplementary Material 2.

In addition, in all the questionnaires (Round 1 to 3) participants were asked to select their department and the size of their municipality (village/small town vs. medium/large city) and self‑rate their knowledge of the issue on a 1–5 scale (1 = no knowledge; 5 = perfect knowledge). The questionnaire was tested internally within the WG in September, 2024. A small group of two national experts per stakeholder category then piloted the questionnaire and their feedback led to wording adjustments to enhance accessibility and specification of risks. These experts were not involved thereafter. To ensure accessibility, the questionnaire was adapted to Easy‑to‑Read standards (FALC) in December 2024 by an independent group composed of older adults trained to the Easy-to-Read method. The group unanimously validated the final questionnaire prior to distribution.

### Round 2

All open-ended responses collected in Round 1 were exported verbatim and analyzed using an inductive content analysis approach. Responses were first segmented into discrete meaning units, each corresponding to a single idea related to a fall risk factor, a preventive action, or a barrier. These meaning units were then iteratively grouped into preliminary themes based on semantic proximity and conceptual similarity. To support this initial structuring, AI-assisted text processing (ChatGPT-4, OpenAI) was used to identify recurrent patterns, cluster similar statements, and generate draft formulations of propositions. This AI-generated output was used exclusively as an aid to organization and did not replace human judgment. Each original response was systematically checked by the research team against the proposed items to ensure that all participant contributions were represented, that wording remained faithful to participants’ intent, and that no new concepts were introduced. During this human validation phase, propositions were reworded for clarity, merged when overlapping, or split when conceptually distinct. No proposition was retained unless it could be explicitly traced back to at least one participant contribution. This approach aligns with recent practice on the use of AI to support qualitative analysis [24–26]. Disagreements were resolved through discussion within the working group until consensus was reached. This process resulted in a consolidated list of propositions that comprehensively reflected the content of Round-1 responses and was subsequently submitted for quantitative rating in Round 2.

Round 2 allowed for open comments in order for the panelists to indicate if their initial propositions were not appropriately transferred in this round. Propositions were presented to WG members at the end of March 2025 and questions were revised collectively. For each item, perceived relevance was rated on a 10‑point Likert scale (1 = not at all important; 10 = extremely important) [17]. The survey was emailed in mid‑May and remained open for three weeks, with a reminder one week before closing.

At the end of the second round, items were classified as: (a) consensual: at least 70% of participants rated an item ≥ 7 (≥ 70%≥7), and the interquartile range (IQR) was ≤ 2.5 resulting in items being selected and not re‑rated in the third round; (b) non‑consensual: <70% ≥7 and/or IQR > 2.5 resulting in items being re‑rated in phase three in light of the results of the second round [19, 21] with possible modification if the panelists suggested them in the open comment section. The criterion was used to capture both a high level of agreement and an acceptable dispersion of ratings, in line with common Delphi practice [19, 21].

### Round 3

In addition to the non-consensual items from round 2 (presented to all participants with median scores and reason for not being selected directly in round 2) new items were introduced in phase three based on round‑two comments. The third questionnaire did not allow for open comments. It was emailed in the last week of June. Phase three remained open for three weeks, with a reminder one week before closing.

### Final analysis and classification of selected items

Levels of knowledge about falls (ordinal scale 1–5) were analyzed using an ordinal logistic regression (cumulative link model). The main predictor was expert group (Seniors, Urban planning, Health and science, Decision-makers), while survey phase was included as a covariate to control for potential variability across rounds. Post-hoc pairwise comparisons between groups were conducted using estimated marginal means with Bonferroni correction for multiple testing. Results were reported as odds ratios (ORs) with 95% confidence intervals (CI).

For each selected item, either validated in the second round or sent to the third, the percentage of respondents scoring ≥ 7, the IQR, and the median were calculated for each group and overall. To allow prioritization, items were classified by median into four perceived‑relevance levels: Median < 7: low relevance; Median ≥ 7: moderate relevance; Median ≥ 8: high relevance; Median ≥ 9: very high relevance. Consensus was considered reached if at least 70% of responses were ≥ 7 and if the IQR was ≤ 2.5. Total consensus meant all groups met these thresholds, global consensus referred to the overall sample meeting them; partial consensus referred to only some expert groups meeting them. Because expertise in fall prevention can span multiple domains, some participants were assigned to more than one expert group and contributed to each relevant group-specific consensus analysis; however, each participant was counted only once in the overall (global) consensus calculations.

## Results

**Fig. 1:**
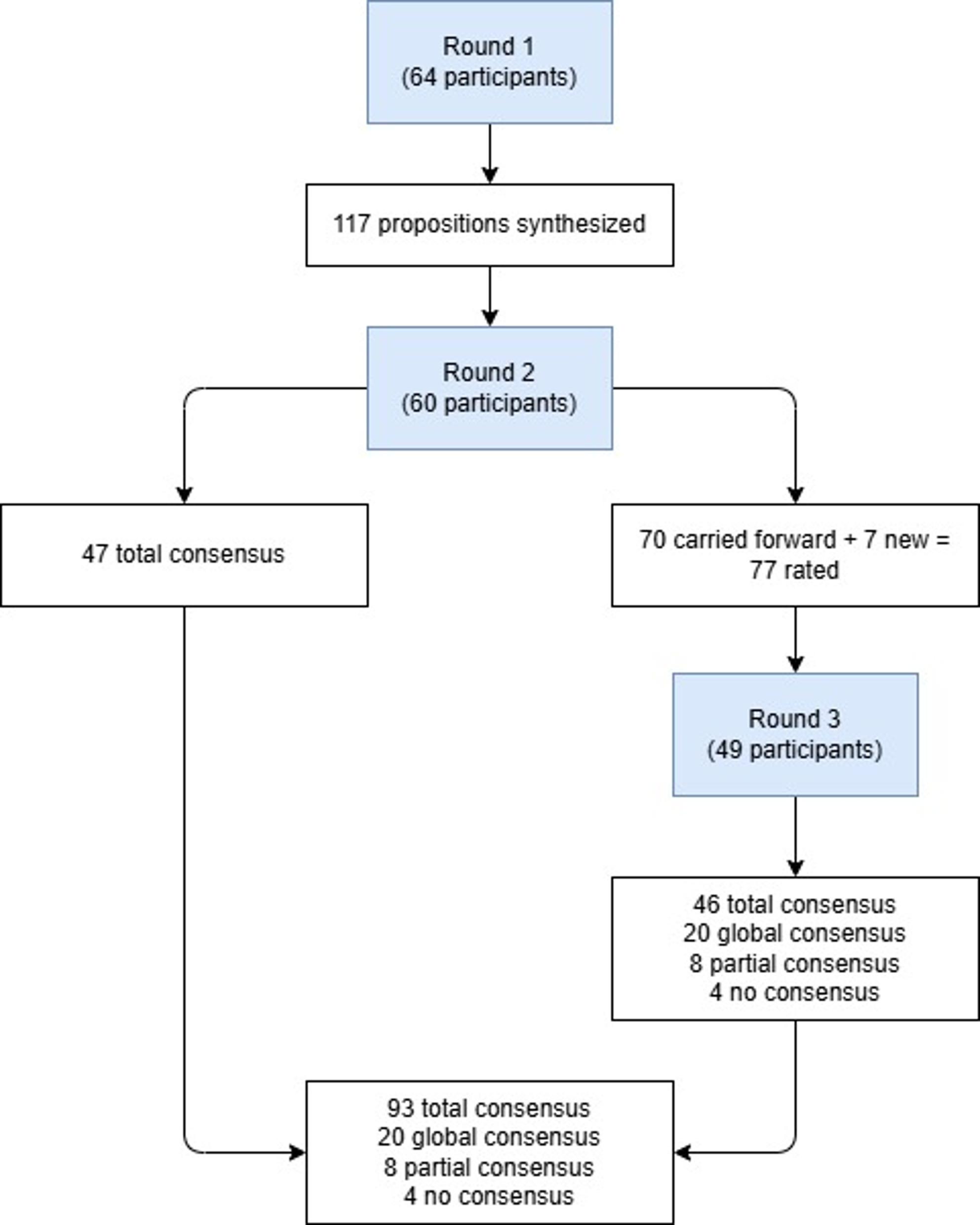
Flow of the three-round Delphi process, showing participant numbers and consensus outcomes

The flow of participants and consensus outcomes across the three Delphi rounds is presented in Fig. 1; Table 1 provides an overview of Delphi panel participation across the three rounds. Results from each Delphi round are presented using a consistent thematic structure across rounds, namely intrinsic factors, extrinsic factors, preventive actions, and barriers.

**Table 1:** Overview of Delphi panel participation across rounds by expert group. Some panelists held multiple areas of expertise and therefore contributed to more than one expert group in group-specific analyses; however, each participant was counted only once in the total number of participants per round

Expert group	Round 1	Round 2	Round 3
Older adults	19	13	13
Health & science	19	28	20
Urban planners	19	9	10
Decision-makers	16	13	13
Total	64	60	49

### Round 1

A total of 117 propositions emerged after combining and refining with AI pre-analysis, research team analysis, and working group comments. The number of items generated in Phase 1 by each group and for each category is presented in Table 2. Four main categories emerged: person-related factors, environmental factors, proposed actions, and barriers to change.

**Table 2:** Number of items generated in Phase 1 by expert group and category

Category	Older adults	Health & science	Urban planners	Decision-makers
Person-related factors	61	124	75	46
Environmental factors	86	107	129	83
Proposed actions	37	78	82	51
Barriers to change	13	21	13	9
Total	197	330	299	189

### Round 2

In phase two, consensus was reached for 47 of the 117 propositions. The remaining propositions were carried into phase three. Four items were reformulated following feedback. For example: the statement “Being a woman strongly influences outdoor fall risk” failed to reach consensus; it was reframed as “Sex has no effect on outdoor fall risk.” The statement “Personal fall‑risk factors outdoors are the same as at home” was reframed as “Personal fall‑risk factors outdoors are specific (and differ from those at home or in institutions).” The statement “Develop contextual signage to dynamically alert pedestrians to temporary hazards (e.g., ice, obstacles, construction)” became “Develop contextual signage, including targeted weather alerts for older adults, to prevent temporary hazards such as ice, obstacles, or construction.” Finally, the statement “Design features perceived as reserved for older people or stigmatizing (e.g., ‘senior’ pictograms) may hinder acceptance” was reframed as “Features perceived as reserved for older adults or stigmatizing (e.g., ‘senior’ pictograms) may hinder their use.” In line with participant suggestions, seven new items were also added in phase three.

### Round 3

**Fig. 2:**
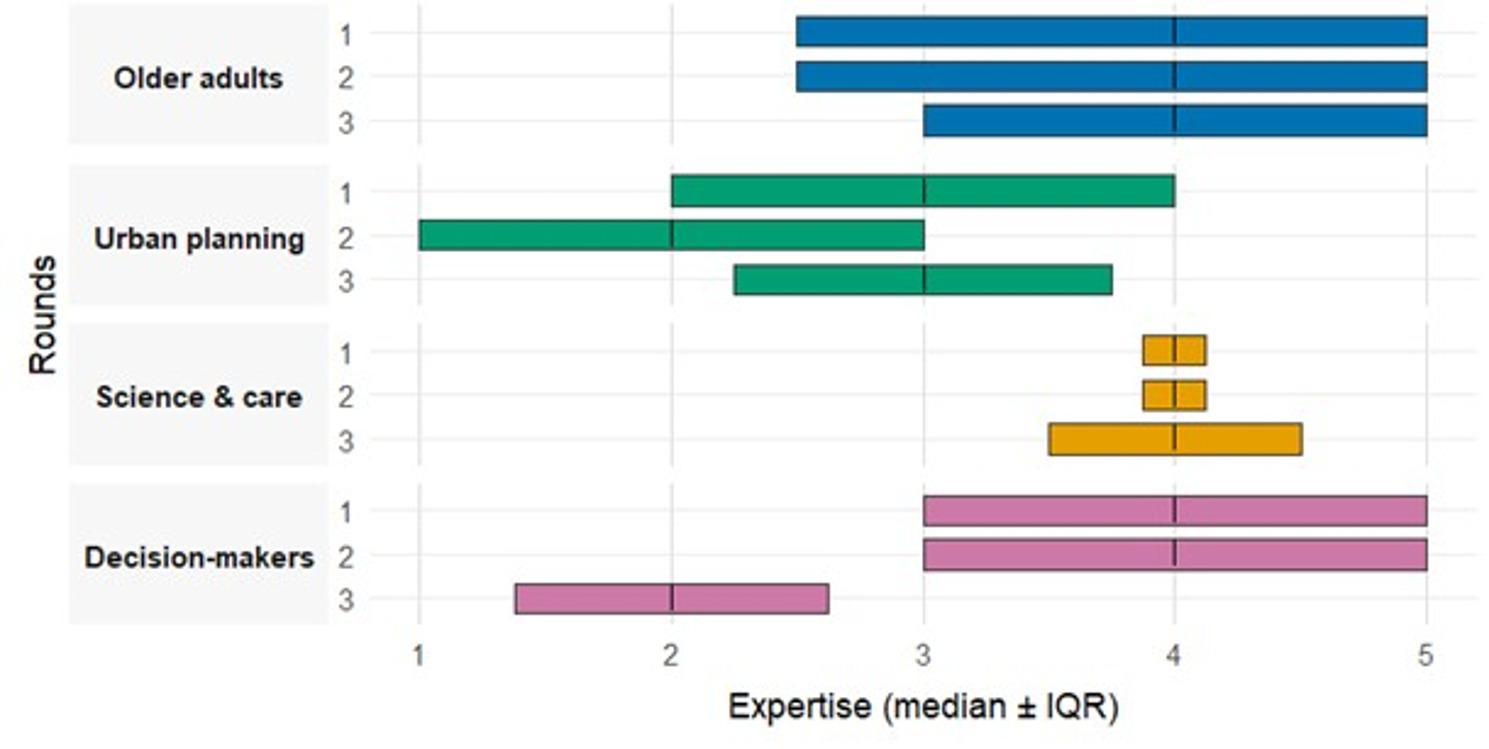
Self-reported expertise across Delphi rounds. Presented as median ± interquartile range (IQR) for each stakeholder group (older adults, urban planning, science & care, decision-makers)

**Fig. 3:**
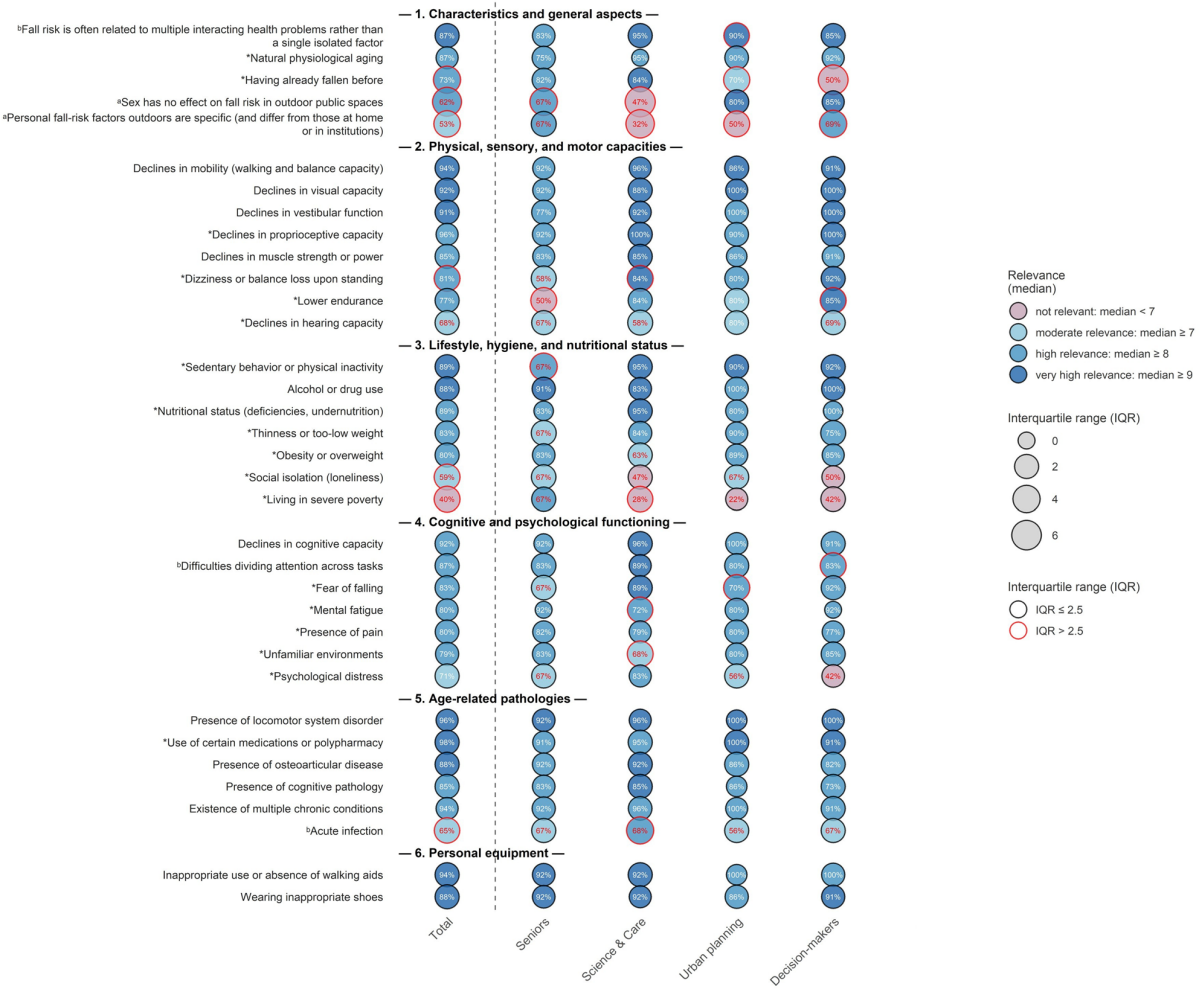
Bubble heatmap of Delphi consensus across expert groups on intrinsic risk‑factor propositions for outdoor public spaces. Bubble fill color indicates relevance level, bubble size the interquartile range (IQR), and text the percentage of ratings ≥7. Red bubble outlines mark high dispersion (IQR >2.5), and red text indicates lack of consensus (<70% ≥7). * = item re-rated in round 3; ᵃ = item modified between rounds 2 and 3; ᵇ = item added in round 3

**Fig. 4:**
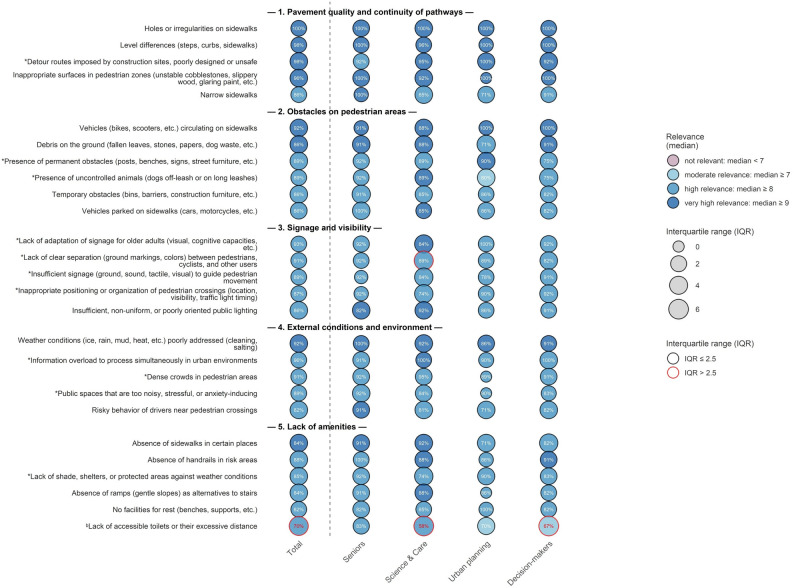
Bubble heatmap of Delphi consensus across expert groups on extrinsic risk‑factor propositions for outdoor public spaces. Bubble fill color indicates relevance level, bubble size the interquartile range (IQR), and text the percentage of ratings ≥7. Red bubble outlines mark high dispersion (IQR >2.5), and red text indicates lack of consensus (<70% ≥7). * = item re-rated in round 3; ᵃ = item modified between rounds 2 and 3; ᵇ = item added in round 3

The final phase included 77 items. Final results are shown in Figs. 1, 2, 3 and 4, in addition to items that reached consensus in phase two.

The analysis of the difference in expertise regarding the issue addressed by this study revealed a significant effect of group on self-reported knowledge levels (χ²(3) = 31.1, *p* < 0.001), while the effect of survey round was not significant (χ²(2) = 0.85, *p* = 0.65). Compared to other groups, participants in the Health and science group reported substantially higher expertise. Specifically, they had approximately eight times higher odds of reporting greater knowledge compared to participants in Urban planning (OR = 8.0, 95% CI [3.4–19.0], *p* < 0.001), 3.4 times higher odds compared to Seniors (OR = 3.4, 95% CI [1.5–8.1], *p* = 0.027), and 9.2 times higher odds compared to Decision-makers (OR = 9.2, 95% CI [3.7–22.9], *p* < 0.001). No other between-group differences were statistically significant.

#### Intrinsic factors

Figure 3 presents the results for intrinsic factors. In *Characteristics and general aspects*, 1 item reached total consensus, 1 global consensus, 2 remained partial, and 1 did not reach any consensus in any group. In *Physical*,* sensory*,* and motor capacities*, 5 items obtained total consensus, 1 reached global consensus, and 2 remained partial. In *Lifestyle*,* hygiene*,* and nutritional status*, 2 items reached total consensus, 3 global, and 2 no-consensus. In *Cognitive and psychological functioning*, 2 items achieved total consensus and 5 global consensus. In *Age-related pathologies*, 5 items reached total consensus, and 1 did not reach consensus. Finally, in *Personal equipment*, 2 items obtained total consensus.

#### Extrinsic factors

Figure 4 presents the results for extrinsic factors. In *Pavement quality and continuity of pathways*, 5 items reached total consensus. In *Obstacles on pedestrian areas*, all 6 items reached total consensus. In *Signage and visibility*, 5 items reached total consensus, and 1 global consensus. In *External conditions and environment*, all 5 items reached total consensus. Finally, in *Lack of amenities*, 5 items reached total consensus and 1 partial consensus.

#### Preventive actions

**Fig. 5:**
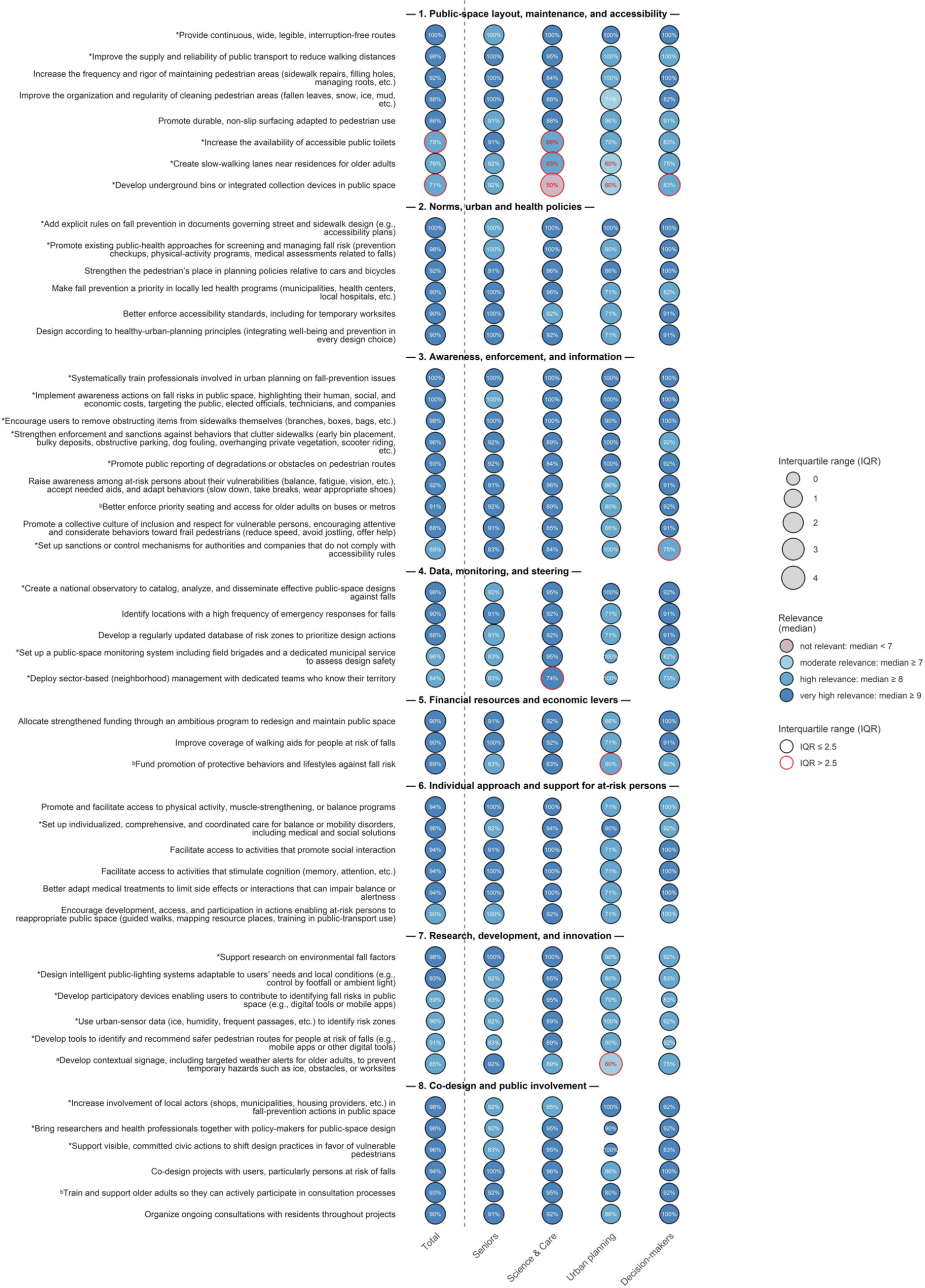
Bubble heatmap of Delphi consensus across expert groups on propositions for preventive actions for falls in outdoor public spaces. Bubble fill color indicates relevance level, bubble size the interquartile range (IQR), and text the percentage of ratings ≥7. Red bubble outlines mark high dispersion (IQR >2.5), and red text indicates lack of consensus (<70% ≥7). * = item re-rated in round 3; ᵃ = item modified between rounds 2 and 3; ᵇ = item added in round 3

Figure 5 presents the results for action propositions. In *Public-space layout*,* maintenance*,* and accessibility*, 5 items reached total consensus, 1 global consensus, and 2 partial consensus. In *Norms*,* urban and health policies*, 6 items achieved total consensus. In *Awareness*,* enforcement*,* and information*, 8 items reached total consensus and 1 global consensus. In *Data*,* monitoring*,* and steering*, 4 items reached total consensus and 1 global consensus. In *Financial resources and economic levers*, 2 items achieved total consensus and 1 partial consensus. In *Individual approach and support for at-risk persons*, 6 items reached total consensus. In *Research*,* development*,* and innovation*, 5 items achieved total consensus and 1 global consensus. Finally, in *Co-design and public involvement*, 6 items reached total consensus.

#### Barriers to preventive actions

**Fig. 6:**
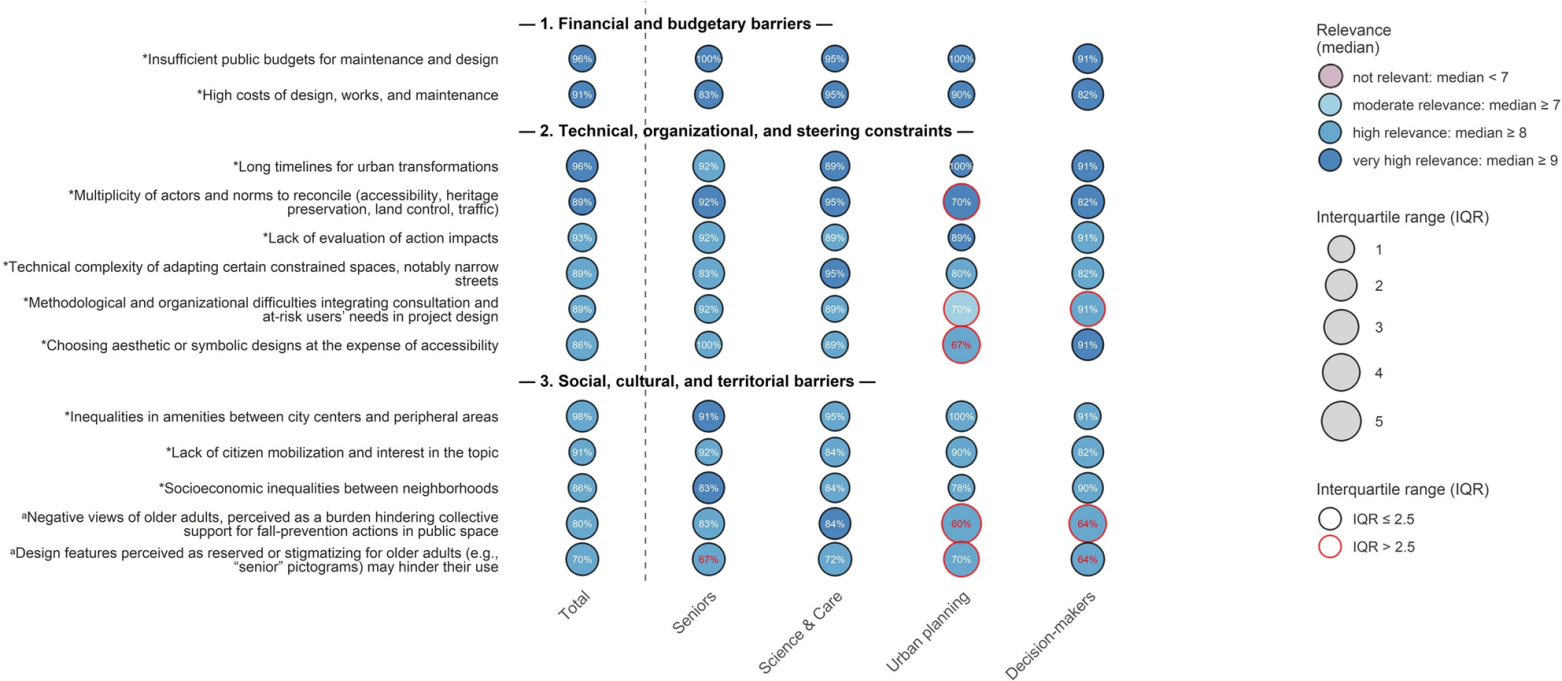
Bubble heatmap of Delphi consensus across expert groups on propositions for barriers to preventive actions. Bubble fill color indicates relevance level, bubble size the interquartile range (IQR), and text the percentage of ratings ≥7. Red bubble outlines mark high dispersion (IQR >2.5), and red text indicates lack of consensus (<70% ≥7). * = item re-rated in round 3; ᵃ = item modified between rounds 2 and 3; ᵇ = item added in round 3

Figure 6 presents the results on barriers to preventive actions. In *Financial and budgetary barriers*, both items reached total consensus. In *Technical*,* organizational*,* and steering constraints*, 3 items achieved total consensus and 3 reached global consensus. In *Social*,* cultural*,* and territorial barriers*, 3 items reached total consensus, while 2 achieved global consensus.

#### Risk–intervention correspondence

**Fig. 7:**
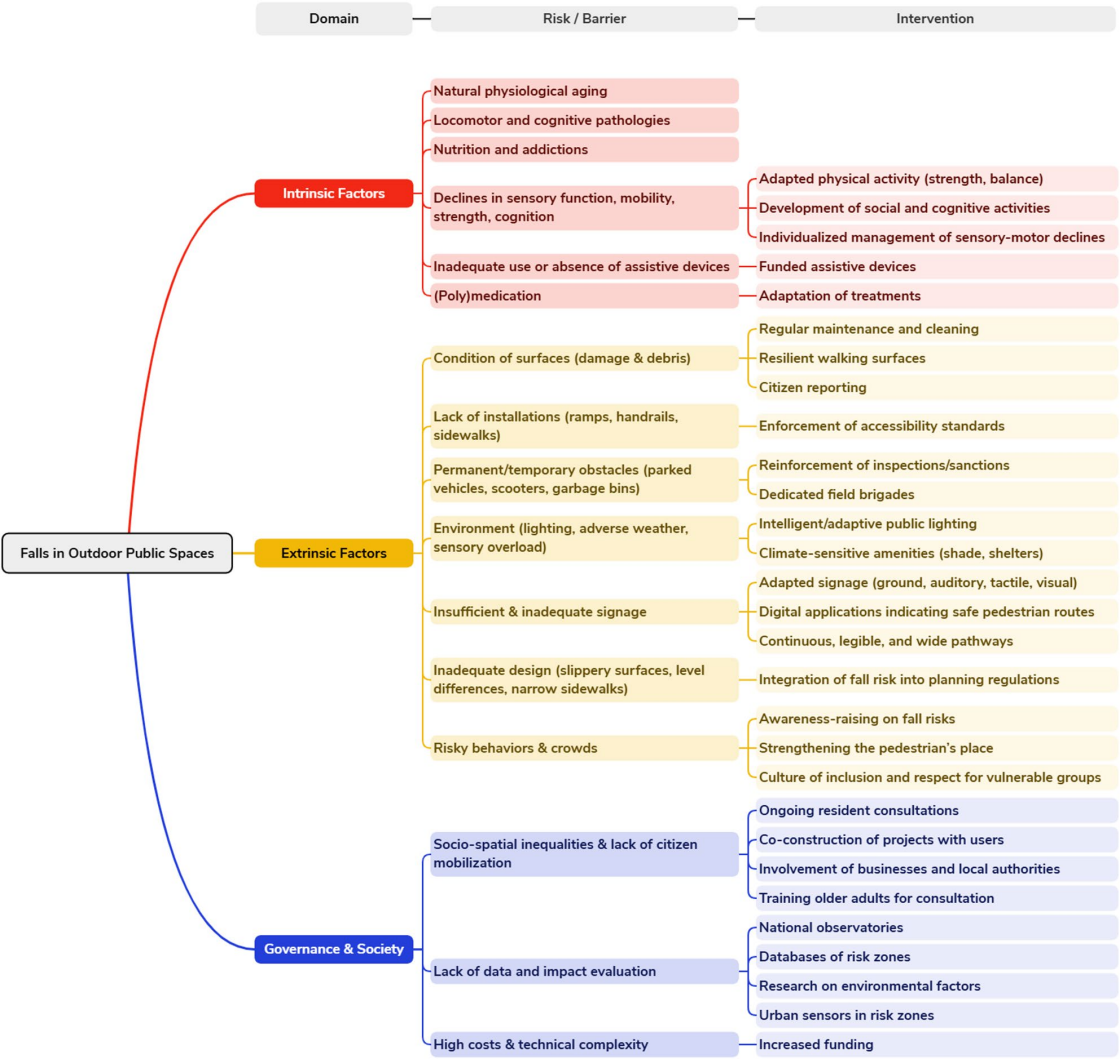
Intrinsic, extrinsic, and governance-related factors identified in the Delphi study on falls in outdoor public spaces and their associated possible preventive actions (red = intrinsic factors, yellow = extrinsic/environmental factors, blue = governance and societal factors; darker boxes indicate identified risks or issues, lighter boxes indicate the associated preventive actions)

To facilitate interpretation of the findings, an illustrative correspondence between the main outdoor fall-related risks identified through the Delphi process and the proposed intervention domains was developed by the multidisciplinary working group. This correspondence, presented in Fig. 7, is intended to support readability and translation of results into practice. It does not constitute an additional consensus outcome, as the Delphi design did not include a formal stakeholder-validated linkage between specific risks and specific interventions.

## Discussion

This Delphi study aimed to identify risk factors for outdoor falls among older adults, as well as the most relevant preventive actions and the barriers to their implementation. It addresses a context where scientific knowledge remains scarce and fragmented and where outdoor falls remain understudied [7]. The study relies on a rigorous three‑phase Delphi process, characterized by iterative rounds, anonymous expert ratings, controlled feedback, a predefined definition of consensus [18, 19], and a multidisciplinary panel to build informed consensus useful for urban and public health strategies.

Recent work has proposed a unified framework for mobility in older persons, distinguishing perceived mobility, actual mobility, and locomotor capacity within the WHO Healthy Ageing model [27]. Although behavioral and sociodemographic aspects of mobility were not targeted as standalone domains in the present Delphi study, several such elements emerged organically across intrinsic and extrinsic factors through participants’ contributions, reflecting their cross-cutting role in outdoor fall risk rather than a separate level of analysis.

### Intrinsic factors

Final results show a strong consensus on intrinsic contributors of older adults to outdoor fall risks, with strong consensus on biomedical but also notable divergences on behavioral, psychosocial, and social factors.

The strongest core consensus, rated highly or very highly concerned biomedical factors, including sensory, cognitive, motor, and musculoskeletal declines (gait and balance impairments; visual, vestibular, cognitive, and proprioceptive deficits; and reduced muscular function). These vulnerabilities, documented in the fall literature, form the biomedical core of risk and confirm that biomedical determinants may remain important to explain outdoor falls [28–30]. Pain, chronic disease, locomotor or osteoarticular disorders, as well as polypharmacy also show strong consensus, consistent with prior work [28, 30]. Panelists also unanimously identified inadequate use of assistive devices and inappropriate footwear as major factors, highlighting behaviors and equipment often less emphasized in classic prevention strategies [31]. Some factors were judged relevant by certain groups but not by others. Obesity and thinness divided groups, possibly because they are often approached using BMI, which poorly reflects body composition [32]. Sex effects are a complicated topic. The initial statement that “being a woman strongly influences risk” was rejected in the second round, and the reformulated “no sex effect” failed to achieve consensus suggesting that panelists do not share a unified view. Epidemiological data often show a higher prevalence of falls among women [33], but this may reverse when focusing on outdoor falls [34]. Mobility behaviors, activities, and exposure to urban environments may differ between sexes and help explain these patterns [33].

The relevance of psycho-behavioral factors could differ between groups. It is also noteworthy that older fallers did not perceive concerns about falling as a risk factor, even though the literature describes it as both consequence and determinant of fall risk that can foster activity avoidance and alter gait [35–37]. Older adults may see fear as a cautious behavior intended to secure movement rather than increase vulnerability [38]. While sedentariness was supported by strong global consensus, it was down‑weighted by older adults. The role of physical activity appears complex, and could be both an indirect protective behavior and a direct risk factor, especially since outdoor fallers are often reported to be particularly active [39]. Of interest, social isolation and deprivation, frequently linked to physical‑activity and cognitive declines, were much less recognized by panelists, who tended to prioritize biomedical dimensions [40].

These divergences reflect different priorities (urban planners and decision‑makers focus on practical levers, health professionals on clinical vulnerabilities, older adults on lived experience). They call for policies that tailor messages for each group. They also highlight the specificity of outdoor environments, even though a key point concerned the absence of consensus on whether intrinsic factors are distinct between home and outdoors. The initial statement that “personal risk factors outdoors are the same as at home” failed to reach consensus in round two. The reformulated statement that “personal risk factors outdoors are specific” also failed to secure overall agreement in round three, although older adults were more favorable to specificity while scientific/health professionals expressed more caution, likely reflecting the current limits of evidence and calling for further research on the topic.

In sum, strong consensus supports sensory, cognitive, motor, and musculoskeletal vulnerabilities, along with polypharmacy and inadequate assistive devices or footwear, as priority targets for outdoor fall prevention. Uncertainties remain regarding the specificity of the context (home vs. outdoor), sex, body composition, activity level, psychosocial vulnerabilities, concerns about falling, and cognitive fatigue in outdoor travel. Mixed designs combining epidemiology, real‑world observation, and qualitative analyses are needed. Although the individual-level factors identified in this study closely align with domains described in the World Falls Guidelines [29], they are conceptualized here as context-specific vulnerability modifiers in outdoor public environments rather than as a clinical risk stratification framework, and should therefore be viewed as complementary rather than interchangeable.

### Extrinsic factors

The study reveals broad consensus on most extrinsic factors with 25 out of 27 items reaching total consensus, underscoring the importance of physical obstacles, missing amenities, and the overall sensory environment as determinants of pedestrian safety.

The strongest consensus was regarded physical and organizational obstacles that directly affect pedestrian safety: unsuitable, degraded, or irregular surfacing; level changes; risky detours around worksites; absence of sidewalks; debris; temporary or fixed obstacles; and vehicles parked or moving on sidewalks. Adverse weather conditions also stand out when poorly addressed. Another core of consensus concerns missing amenities that support continuous and secure travel (benches, shelters, ramps, continuous wide sidewalks) and signage that is absent or not adapted to older adults (with possible sensory decline). This shapes a set of risk factors that can be assessed in the environment to determine the risk of falls. The ambient sensory environment (visual overload, crowding, excessive noise) also mattered to the panelists, highlighting that prevention must go beyond physical layout to include overall urban quality and users’ capacity to process information.

These priorities align with WHO “age‑friendly cities” recommendations. Strong consensus on safe, continuous pathways; public lighting; benches and ramps; and securing worksites and temporary obstacles fits the framework’s key dimensions [14]. However, while the WHO framework considers public‑space safety broadly, it does not address falls directly. Our study centers falls explicitly and highlights factors of specific relevance. Adding a specific “fall prevention” axis would increase the framework’s operational relevance for this public‑health challenge.

Our results also overlap with walkability frameworks. Walkability typically addresses environments that support pedestrian movement and sometimes perceived comfort and safety, but it differs markedly from the topic of fall risks in older age, especially because many classic indices are not adapted to aging [41]. Even in older‑adult‑oriented studies, the focus is rather on physical activity, social participation, or daily mobility, and “falls” is absent despite their public‑health importance [41]. This study therefore complements existing frameworks by confirming shared criteria while enlarging evaluation to vulnerabilities specific to older fallers.

### Preventive actions

This present Delphi study support that effective prevention of outdoor falls should not solely focus on fixing sidewalks and physical environment, but is instead a multiaxial challenge. While walking surfaces and their quality are priorities often cited in participatory studies [42–44], the present study provides a broader set of preventive actions. These can be organized into: regulation and governance, awareness and education, monitoring and technology, preventive action targeting intrinsic factors, and social and participatory involvement.

A particularly strong consensus concerns embedding fall prevention within regulatory and policy frameworks, beyond clinical settings. Panelists called for dedicated funding via ambitious programs and explicitly integrating fall prevention into planning documents and local health programs. They advised better enforcement of existing accessibility norms, and a strengthening the pedestrian’s place relative to cars and bicycles, in line with broader shifts away from car‑centric mobility [45, 46].

Awareness, training and information are also seen as highly relevant, with most related items reaching total consensus. Broad public campaigns on outdoor fall risk gained strong support, as did tailored awareness for at‑risk individuals to recognize vulnerabilities and adapt behaviors, in line with research showing the importance of self-efficacy in fall prevention [36]. A novel, strongly supported point is systematic training of urban‑planning professionals on fall prevention. At the interface of regulation and awareness, stronger controls and sanctions against cluttering sidewalks (with scooters, parking.) are supported, indicating that information alone is insufficient without enforcement. Promoting a culture of respect for vulnerable pedestrians was also consensual among panelists and promotes the need for cultural change in line with previous identified top priority for age friendly cities [47]. Practical measures such as systematic reporting of obstacles, encouraging users to remove minor obstructions, and priority seating for older adults in transit illustrate a balance between individual, collective, and institutional responsibility proposed by the panelists.

Proposals for action involving technological innovations also occupy a notable place. The use of technologies is increasingly considered by experts as a possible solution to address some of the challenges associated with urban aging, but remains underexploited [48]. Experts propose citizen‑reporting apps to alert municipalities about hazards. Existing initiatives seldom target fall risk specifically and are not widely deployed [49]. Using urban sensors to detect conditions (degradation, weather, crowding) and deploying adaptive public lighting were also proposed. While older adults are often thought to be wary of technology in relationship with personal, technical and contextual factors [50] participants in our study, especially older adults, were among the most favorable to such solutions. Strong consensus also supported better structuring of data production and use in relationship with fall prevention. A national observatory to catalog, analyze, and disseminate effective interventions demonstrate the need for evidence‑based steering. Identifying hotspots of emergency responses to falls and maintaining updated risk‑zone databases can also help locate where to act first. This aligns with calls to strengthen data governance in public health and urbanism and with the international trend toward using spatial data to target urban vulnerability [51, 52]. Participants also strongly recommended supporting research on environmental contributors to falls.

Panelists agreed on the need for individualized, coordinated care for mobility and balance disorders, integrating medical, social, and environmental dimensions. Proposed innovative actions with broad support include enabling at‑risk persons to reappropriate public space (guided walks, training for transit use…), which can reduce anxiety and foster autonomous mobility [53–55]. Social participation and access to group activities, also consensual among panelists, can also reduce isolation and support mental health [56, 57] and may indirectly contribute to fall prevention. However, their effectiveness warrants further study. Access to targeted physical activity programs (balance, strength) is rated as highly pertinent and is among the best‑established primary and secondary prevention domains [58]. Finally, experts emphasize better consideration of cognitive and medication factors, including optimized prescriptions to limit side effects and interactions, in line with guidance on fall‑risk‑increasing drugs [59].

Finally, a very strong consensus supports embedding fall prevention within co‑construction approaches. Essential dimensions include linking researchers and health professionals with policy‑makers for public‑space decisions, co‑constructing projects with users (especially those at risk), and organizing continuous consultation with residents. This aligns with international movements in citizen science and participatory urbanism, which improve intervention fit to real needs [60]. Involving diverse publics (older adults, urban planners, local decision‑makers) can be especially effective for promoting environments that support physical activity and social interaction [61]. Because the present results emerged from a structured, participatory process involving multidisciplinary experts and users, they can be considered particularly legitimate. They illustrate that falls, too often framed as a clinical issue, are a collective challenge requiring novel alliances among public health, urban planning, and civil society [62]. Panelists also stress the role of local actors of proximity (shops, landlords, municipalities), whose involvement can shift practices toward safer environments for older pedestrians. Training and supporting older adults to participate actively in consultation processes recognizes them not only as beneficiaries but as experts in using public space, consistent with empowerment approaches [63]. The strong consensus behind these actions is decisive for deployment as initiatives supported across users, professionals, and decision‑makers are better implemented and accepted [64].

### Barriers to preventive actions

The Delphi results highlight that the relevance of barriers to preventive actions may differ among expert groups, with strong consensus on financial obstacles but more divergence on technical, organizational, and social dimensions. The study findings regarding implementation barriers are consistent with those reported in a recent systematic review on community fall prevention implementation [65], particularly with respect to resource constraints, governance fragmentation, and challenges in intersectoral coordination. This DELPHI study extends these insights beyond exercise program implementation to the context of outdoor public spaces, highlighting similar structural barriers across health, urban planning, and governance domains.

Financial barriers were the most consensual. High costs of design and maintenance and insufficient dedicated public funding were unanimously identified as major obstacles. This convergence reflects a well‑documented reality: adapting urban environments to older populations’ needs is often seen as secondary to other municipal priorities [66–68]. Our results argue for embedding aging needs in urban planning and for stable resources, as promoted in age‑friendly city programs.

Technical and organizational constraints were less consensual. The lack of systematic impact evaluation is a key barrier with consensus, echoing prior work showing that many age‑friendly initiatives lack rigorous evaluation, leaving a fragmented evidence base [48]. Without strong data on effects, sustaining investment is difficult, even when interventions seem promising [69]. Stakeholders themselves emphasize the need for standardized frameworks and tools to evaluate and compare interventions [70]. Developing shared instruments and continuous validation cycles appears critical to strengthen legitimacy and facilitate integration into policy [71]. In the specific context of outdoor falls, although fall risk is sometimes cited as something to assess, there are no specific environmental evaluation tools [72]. Evaluations could rely on this Delphi’s results and combine quantitative indicators (fall and hospitalization rates), qualitative indicators (sense of safety, avoidance behaviors), environmental indicators (maintenance, lighting, signage), and governance indicators (budgets, citizen‑reporting mechanisms).

While participants agreed on the technical complexity of adapting constrained spaces and on long timelines, other aspects are debated. Methodological and organizational hurdles to participatory processes did not reach total consensus. Urban planners may be more familiar with participatory devices (workshops, public inquiries, mandated consultations) than other participants assume, and thus view these as part of routine practice rather than major barriers (in comparison with more structural barriers like financial constraints [70], whereas others may perceive them as significant obstacles to co‑construction.

Panelists broadly agree that territorial (e.g., center vs. periphery) and socioeconomic inequalities between neighborhoods is a barrier to effective prevention. These disparities may reflect unequal distribution of amenities conducive to “aging well” [73]. Evidence directly linking such inequalities to fall risk is limited, though more favorable neighborhood environments for leisure physical activity may reduce falls among older adults [74]. Lack of citizen mobilization and public interest was also reported as a pertinent barrier, possibly reflecting limited public awareness of the issue, recognized as a key challenge for implementing age‑friendly environments [66]. By contrast, the impact of negative social opinion of aging is disputed: older adults often identify ageism as a barrier to active aging, whereas other actors may down‑weight it [75]. The fact that elected officials and urban planners did not rate this barrier as a priority could reflect lower perceived importance or overestimation by older adults.

Although established implementation frameworks such as CFIR and TICD offer valuable structures for analyzing determinants of implementation, the present study adopted an inductive, functionally oriented classification of barriers to better reflect the cross-sectoral nature of outdoor fall prevention and to avoid constraining stakeholder input within predefined health-centered categories [76, 77].

### Recommendations

Based on these results and the literature, several recommendations can guide public policies and practices for preventing outdoor falls (Fig. 7). Falls should no longer be considered only a clinical problem; they are a transversal issue of public health and urban planning. Municipalities should explicitly embed fall prevention in urban, mobility, and health plans with stable dedicated funding [14, 66]. Strong consensus highlights basic pedestrian safety dimensions as immediate, widely shared levers to reduce fall risk. Investing in systematic maintenance protocols, rapid repairs, and securing risk zones should become standard practice. Multidisciplinary actions (systematic inclusion of fall prevention in planning documents, targeted training for planners and technical agents, and monitoring tools such as observatories and participatory reporting) should also be prioritized.

The results support that prevention requires shared governance mobilizing researchers, professionals, decision‑makers, associations, and citizens. Consultations should be regular, inclusive, and adapted (Easy‑to‑Read, digital tools, exploratory walks), giving older adults their place as expert users while also engaging other citizens so the issue becomes collective [60, 61]. Public awareness campaigns should reinforce recognition of falls as a shared concern, not one reserved to older people. Involving local proximity actors (shopkeepers, landlords, associations) could help diffuse a common culture of safety [62]. Prevention must prioritize peripheral and disadvantaged neighborhoods, where infrastructure is often less developed and risks are increased by social and spatial conditions.

A major barrier, beyond funding, is the lack of systematic evaluation. It is crucial to develop standardized instruments specific to outdoor fall risk, combining quantitative indicators (falls, hospitalizations), qualitative indicators (risk perception, sense of safety), environmental indicators (maintenance, lighting, signage), and governance indicators (budgets, participatory mechanisms). Such tools would enable comparisons, objectify progress, and strengthen legitimacy of investments [70, 71]. Pilot projects deploying innovative solutions (connected technologies, intelligent signage, participatory platforms) should be encouraged, with attention to accessibility for older users.

### Strength, limitations and perspectives

Beyond the under‑studied topic of outdoor falls, a key originality of the study is the multidisciplinary panel including: older adults who have already fallen outdoors, scientific experts and health professionals, urban planners, and decision‑makers. Delphi studies relatively rarely include populations directly affected (27% according to Schifano & Niederberger, 2025). Their inclusion here allows us to benefits from their lived experience as expertise while ensuring knowledge sharing from technical, scientific, and policy views. This diversity yielded complementary and sometimes contrasting results, enabling shared priorities to emerge while identifying areas of divergence. The methodology followed recent recommendations [19], maintained a retention rate consistent with standards (− 18.3% from first to last phase), and applied strict consensus criteria (≥ 70% ratings ≥ 7/10 and IQR ≤ 2.5). The combined use of an initial AI‑assisted synthesis with systematic human validation for the open qualitative responses in round one is innovative and aligns with guidance on limiting bias [24, 25]. Adapting the questionnaire to Easy‑to‑Read standards broadened inclusion of older profiles, including those less familiar with research, enhancing panel representativeness.

This study has several limitations. First, it was conducted in Normandy, France, and although the region is demographically and urbanistically representative of many European regions undergoing advanced demographic transition, priorities and perceptions may vary in other contexts, such as very dense urban areas or countries with different infrastructures and policies. Replication elsewhere is needed to test transferability. Choosing a very broad geographical area (multiple countries) could have led to a consensus emerging only on very general aspects, more widely applicable but less insightful. Second, panel recruitment was voluntary, which may introduce selection bias toward more sensitized stakeholders. Complementary population surveys or random panels could enhance representativeness. In addition, although the targeted minimum number of urban planners was not reached in Round 2 (*n* = 9), this threshold was reached again in Round 3; nevertheless, urban planner–specific agreement in Round 2 should be interpreted with caution, without affecting the validity of the overall panel-level consensus. Third, recent work has proposed a unified framework for mobility in older persons in which behavioral and sociodemographic dimensions are integral components shaping mobility [27]. Although these aspects were not targeted as standalone domains in the present Delphi design, several behavioral and sociodemographic elements emerged across intrinsic and extrinsic factors through participants’ contributions, reflecting their influence on outdoor fall risk. Future studies should specifically examine behavioral and sociodemographic dimensions of mobility and their interactions with outdoor environments in relation to fall risk. Fourth, as with any Delphi, results reflect perceptions and expertise rather than objective measurement, and they have not yet been matched to field data (epidemiological, sensor‑based, in situ observation). Developing standardized evaluation tools that combine quantitative, qualitative, environmental, and governance indicators would strengthen external validity. Fifth, consensus methods inherently produce compromises. Technical details (e.g., precise surfacing specifications) or very personal issues (e.g., anxiety after a fall) may have been diluted in the final ranking even if recognized as important in the literature. In‑depth qualitative interviews, exploratory walks, and in situ observations could identify latent needs or “blind spots,” refining understanding of still understudied dimensions (e.g., sensory overload, cognitive fatigue, combined environmental conditions). Finally, pilot tests with longitudinal evaluation are needed to test effectiveness and reproducibility across contexts. Embedding these priorities in local public‑health and urban‑planning policies, with shared governance and stronger citizen mobilization, could be decisive in sustainably reducing outdoor fall risk.

## Conclusions

Fall-prevention guidelines mainly address clinical aspects of fall prevention without addressing risks in outdoor public spaces, while age-friendly frameworks promote accessibility without considering falls. Outdoor fall prevention thus remains neglected, and this Delphi study helps fill that gap by documenting risks, actions, and barriers in public spaces, and by showing that effective strategies require bridging clinical and environmental perspectives through shared governance. Building prevention as a transversal issue across health and urban planning is key to safer and more inclusive environments for older adults.

## Supplementary Information

Supplementary Material 1.

Supplementary Material 2.

## Data Availability

The data that support the findings of this study are available from the corresponding author upon reasonable request.
