# Supplementary material for: Falls prevention for older adults in outdoor public spaces: an interdisciplinary Delphi consensus on risks, actions, and barriers
Source: BMC Public Health. 2026 May 2;26:1945. doi: 10.1186/s12889-026-27548-1 (PMC13289449; doi:10.1186/s12889-026-27548-1)
Supplement: Supplementary file 1 — Supplementary Material 1. [file 12889_2026_27548_MOESM1_ESM.pdf]

LimeSurvey

# You may now proceed to the actual questionnaire by clicking "Next."

## Section A: Creation of Your Pseudonym

How does one create an individual code to safeguard one's responses?

**A1. What are the first two letters of your place of birth?**

*Example: PA for Paris*

**What are the first two letters of your given name ?**

*Example: AN for Antoine*

**Please indicate the two digits corresponding to your day of birth.**

*Example: 07 for June 7, 1952*

*In this example, the code to be entered would therefore be:* PAAN07

## Section B: Questions about yourself:

**B1. To which category of subject matter expert do you belong? (multiple answers possible)**

- Individuals aged 65 or older who have previously experienced a fall in an outdoor public space. ☐
- Professional possessing scientific expertise regarding fall risk. ☐
- Professional in planning or urban development (including technical services within a local authority). ☐
- Professional involved in the care or emergency response for individuals aged 65 or older who have fallen. ☐
- Representative of a local authority (elected official, territorial agent, etc.) who has previously participated in decision-making related to planning or urban development. ☐

LimeSurvey

**B2.** **Where do you reside?**

- Village or small town ☐
- Medium-sized or large city ☐

**B3.** **Where are you employed?**

- Village or small town ☐
- Medium-sized or large city ☐

## B4. In which department do you reside?

- Calvados (14) ☐
- Eure (27) ☐
- Manche (50) ☐
- Orne (61) ☐
- Seine-Maritime (76) ☐

## B5. In which department do you work?

- Calvados (14) ☐
- Eure (27) ☐
- Manche (50) ☐
- Orne (61) ☐
- Seine-Maritime (76) ☐

**B6.**

| | 1 | 2 | 3 | 4 | 5 |
|---|---|---|---|---|---|
| On a scale from 1 to 5, how would you rate your level of knowledge regarding falls? (1 = No knowledge at all, 5 = Expert knowledge) | ☐ | ☐ | ☐ | ☐ | ☐ |

# Section C: Risk Factors for Falls

**We will begin by asking you a series of questions regarding the causes of falls. Afterwards, we will request your suggestions for potential solutions.**

**The questions are open-ended, meaning you may provide multiple responses to each question. Should you find any question unclear or difficult to understand, you may choose to skip it.**

**By "outdoor public space," we refer to any area outside buildings, such as roads, sidewalks, squares, crosswalks, bus stops, and parks; this does not include private spaces, transportation, or public buildings.**

**C1.** **What are the causes of falls that you have observed?**

**If you have experienced or witnessed a fall involving an older adult in a public outdoor space*, what, in your opinion, were the contributing factors?**

**By "public outdoor space," we refer to any area outside of buildings, such as roads, sidewalks, squares, pedestrian crossings, bus stops, parks, but not private spaces, transportation, or public buildings.*

**C2.** **Which individual factors increase the risk of falling?**

**In other words: In your opinion, which person-related factors contribute to an increased risk of falling in outdoor public spaces?**

***For example: physical issues such as vision, balance, muscle strength, or health; psychological concerns; problems associated with objects; or other relevant issues you consider significant.***

LimeSurvey

**C3.** **Which personal factors contribute to reducing the risk of falling?**

**That is to say: in your view, which factors related to the individual help lower their risk of falling in outdoor public spaces**
**?**

**C4.** **Which environmental factors <u>increase</u> the risk of falling?**

**That is to say: In your opinion, which factors external to the individual increase the likelihood of falling in outdoor public spaces?**

*For example: elements associated with other individuals, the environment (whether temporary or permanent issues), or concerns related to public space management (such as maintenance of green areas, waste collection, construction sites, ongoing works), or any other relevant matters.*

**C5.** **Which environmental factors <u>help reduce</u> the risk of falling?**

**That is to say: In your opinion, which factors external to the individual contribute to lowering their risk of falling in outdoor public spaces?**
**?**

# Section D: Measures to Implement for the Prevention of Falls in Public Spaces

**D1.** **What modifications to the physical layout could help reduce falls in public spaces?**

**That is, in your opinion, which specific changes to the design of outdoor public spaces could help lower the risk of falls?**

*Examples: Such modifications may involve installing new facilities, undertaking repairs, constructing or setting up either permanent or temporary structures, or any other element you consider relevant.*

**D2.** **What changes to the management of public spaces could help reduce falls?**

**In other words, which specific modifications to the external management of public spaces do you believe could effectively lower the risk of falls?**

*Examples: Such changes may involve preventive measures, awareness campaigns, planning, monitoring, or management of traffic and construction zones. They may also include the involvement of stakeholders, mechanisms for reporting risks, or any other aspect you consider relevant.*

**D3.** **What behavioral changes could help reduce falls in public spaces?**

**In other words, in your opinion, what specific behavioral adjustments within public spaces could lessen the risk of falling?**

*Explanation: These actions may be undertaken either individually or collectively. They may pertain to the individual who falls or to those around them.*

**D4.** **How would you characterize an ideal public space that is free from the risk of falls?**

**In other words: In your view, what features constitute an 'ideal public space free from the risk of falls'?**

*Examples: Please describe what you consider most effective in preventing falls. You may provide specific examples of measures already implemented or locations that serve as sources of inspiration.*

## Section E: Barriers and Facilitators

**E1.** **In your opinion, what challenges may arise in the implementation of the actions you have identified?**

**E2.** **In your view, what factors could facilitate the development or wider adoption of the actions you have mentioned?**

# Section F: Final Questions

**F1.** **Do you have any additional comments regarding the risk of falling in outdoor areas?**

*Explanation: You may include any information that you were unable to convey through the previous questions or address topics that you consider important and that have not been discussed.*

**F2.** **Do you have any comments regarding this questionnaire?**

**This questionnaire has been completed. Your responses have been submitted.**

**You will soon receive a second questionnaire. This subsequent questionnaire will address the topics raised in the first.**

**Your response is essential to ensure the smooth progress of this survey.**

**Thank you very much for your participation.**
