# Supplementary material for: Falls prevention for older adults in outdoor public spaces: an interdisciplinary Delphi consensus on risks, actions, and barriers
Source: BMC Public Health. 2026 May 2;26:1945. doi: 10.1186/s12889-026-27548-1 (PMC13289449; doi:10.1186/s12889-026-27548-1)
Supplement: Supplementary file 2 — Supplementary Material 2. [file 12889_2026_27548_MOESM2_ESM.pdf]

DELPHISTAR

Delphi studies in social and health sciences – recommendations for a **sta**ndardized **r**eporting

DeWiss Delphi techniques in health and social sciences

PH Schwäbisch Gmünd University of Education

Delphi studies in social and health sciences – recommendations for an interdisciplinary standardized reporting (DELPHISTAR).

From: Niederberger, M., Schifano, J., Deckert, S., Hirt, J., Homberg, A., Köberich, S., Kuhn, R., Rommel, A., Sonnberger, M. & the DEWISS network (2024). Delphi studies in social and health sciences—Recommendations for an interdisciplinary standardized reporting (DELPHISTAR). Results of a Delphi study. *PLoS ONE 19(8):* e0304651. https://doi.org/10.1371/journal.pone.0304651

More information under OSF (https://osf.io/gc4jk) and DEWISS (https://delphi.ph-gmuend.de/)

Topic	Section	Item	Checklist Item	Location where item is reported	Citation of the text
I<br>Title and Abstract		1	Identification as a Delphi study in the title	(to be adapted with final document)	Falls prevention in Outdoor Public Spaces: An Interdisciplinary Delphi Consensus on Risks, Actions, and Barriers
		2	Identification as a Delphi study in the abstract		A Delphi study was selected to answer the research question.
		3	Structured abstract		Background, methods, results and conclusion
II<br>Context	Formal	4	Information about the sources of funding		No specific funding was received for this Delphi study.
		5	Information about the team of authors and/or researchers (e.g., discipline, institution)		The working group (WG) responsible for this study decided was built within a regional "Anti-Fall Plan" and composed of various stakeholders linked to older adults' fall risk outdoors: older users, urban-planning professionals, physicians and researchers on aging and falls, local decision-makers, and associations.
		6	Information about method consulting		The WG group was advised by experts from XXX regarding the methodology.
		7	Information about the project background		The working group (WG) responsible for this study decided was built within a regional "Anti-Fall Plan"
		8	Information about the study protocol		The University of Caen Normandy Research Ethics Committee approved the study protocol (no. 2025030408142400000260000327).

Delphi studies in social and health sciences – recommendations for an interdisciplinary standardized reporting (DELPHISTAR)

Topic	Section	Item	Checklist Item	Location where item is reported	Citation of the text
	**Content**	9	Justification of the chosen method (Delphi) to answer the research question		Very few scientific studies have so far systematically documented the risk factors for falls among older adults in outdoor public spaces, and they have simply relied on fallers' recollections to identify types of fall risks (Chippendale et al., 2017; Nachreiner et al., 2007; Nicklett et al., 2017). This approach can be affected by recall bias and does not provide actionable solutions for fall prevention. Faced with this lack of consolidated data, the Delphi method appears particularly relevant.
		10	Aim of the Delphi study (e.g., consensus, forecasting)		In this context, the objective of the present study is to document, using an interdisciplinary classic Delphi method, the intrinsic (person-related) and extrinsic (environment-related) risk factors for outdoor falls among older adults, as well as the preventive actions and barriers to their implementation in public spaces.
III Method	**Body & Integration of knowledge**	11	Identification and elucidation of relevant expertise, spheres of experience, and perspectives (e.g., theory, practice, affected groups, disciplines)		They decided to target four categories: (1) older adults aged 65+ who had already fallen outdoors, providing experiential expertise; (2) scientific or technical experts and health professionals, with clinical or research expertise (geriatricians, emergency physicians, GPs, researchers in falls prevention.); (3) planning experts, with professional expertise in the design and management of public space (technical municipal services, urban planners, architects, etc.); and (4) decision-makers with governance expertise (local authority representatives, elected officials).
		12	Handling of knowledge, expertise and perspectives which are missing or have been deliberately not integrated		NA
		13	Basic definition of expert[1]		experiential or professional knowledge of how outdoor public spaces could contribute to fall in older adults

Topic	Section	Item	Checklist Item	Location where item is reported	Citation of the text
	Delphi variant and modifications	14	Identification of the type of Delphi variant and potential modifications (e.g., classic Delphi, real-time Delphi, group Delphi)		using an interdisciplinary classic Delphi method
		15	Justification of the Delphi variant and modifications, including during the Delphi study, if applicable		followed current recommendations for health and social sciences
	Sample of experts	16	Selection criteria for the experts (per round, per expert group if applicable)		Names and emails were proposed by WG members and snowball recruitment was also used as recommended to improve Delphi sampling (Rowe and Wright, 2011). A total of 171 contacts were invited even if they had not responded previously. The target was to collect at least 60 responses in phase 1 and not drop below 47 in the final phase with at least 10 participants per expert group.
		17	Identification of the experts		Names and emails were proposed by WG members and snowball recruitment was also used
		18	Information about recruiting and any subsequent recruiting of experts		An invitation letter was emailed detailing objectives, confidentiality, schedule, and the survey link
	Survey	19	Elucidation of the content development for the questionnaire[2]		The first-round questionnaire was co-designed within a regional 'Anti-Fall Plan' by the 'Outdoor Public Space' working group (WG) composed of various stakeholders linked to older adults' fall risk outdoors: older users, urban-planning professionals, physicians and researchers on aging and falls, local decision-makers, and associations. The objective … was to collect information on risk factors in outdoor public spaces, on possible solutions to reduce these falls, and on potential barriers to these solutions.
		20	Description of the questionnaire (content and structure)		thirteen open questions into three sections: causes of falls; actions to adapt public space; and barriers and facilitators to implementing prevention actions. Causes of falls were explored via five open questions … Actions to prevent falls were explored by four open

Topic	Section	Item	Checklist Item	Location where item is reported	Citation of the text
					questions … Barriers and facilitators were explored by two open questions … Two final open questions captured any information not covered earlier. … In subsequent rounds, propositions were rated on a 10-point Likert scale, with space for open comments in round two
	**Delphi rounds**	21	Number of Delphi rounds		The number of rounds was defined in advance to be a maximum of three rounds.
		22	Information about the aims of the individual Delphi rounds		*“The objective of this first questionnaire was to collect information on risk factors … possible solutions … and potential barriers.” “Open-ended responses from the first round were analyzed to synthesize a limited set of propositions to be rated quantitatively in phase two.” “The final phase included 77 items … non-consensual items from round 2 were re-rated … new items introduced based on round-two comments.”*
		23	Disclosure and justification of the criterion for discontinuation		The number of rounds was defined in advance to be a maximum of three rounds.
	**Feedback**	24	Information about what data was reported back per round		non-consensual: <70% ≥7 and/or IQR > 2.5. The item was re-rated in phase three, in light of the results of the second round (Boulkedid et al., 2011; Schifano and Niederberger, 2025) with possible modification if the panelists suggested them in the open comment section.
		25	Information on how the results of the previous Delphi round were fed back to the experts surveyed (e.g., via frequencies, mean values, measures of dispersion, listing of comments)		presented to all participants with median scores and reason for not being selected directly in round 2
		26	Information on whether feedback was differentiated by specific groups (e.g., by field of expertise, institutional affiliation)		presented to all participants with median scores and reason for not being selected directly in round 2
		27	Information about how dissent and unclear results were handled		non-consensual: <70% ≥7 and/or IQR > 2.5 resulting in items being re-rated

Topic	Section	Item	Checklist Item	Location where item is reported	Citation of the text
					in phase three in light of the results of the second round
	**Data analysis**	28	Disclosure of the quantitative and qualitative analytical strategy		At the end of the second round, items were classified as: (a) consensual: at least 70% of participants rated an item ≥7 (≥70%≥7), and the interquartile range (IQR) was ≤ 2.5 resulting in items beeing selected and not re-rated in the third round; (b) non-consensual: <70% ≥7 and/or IQR > 2.5 resulting in items being re-rated in phase three in light of the results of the second round (Boulkedid et al., 2011; Schifano and Niederberger, 2025) with possible modification if the panelists suggested them in the open comment section. Levels of knowledge about falls (ordinal scale 1–5) were analyzed using an ordinal logistic regression (cumulative link model). The main predictor was expert group (Seniors, Urban planning, Health and science, Decision-makers), while survey phase was included as a covariate to control for potential variability across rounds. Post-hoc pairwise comparisons between groups were conducted using estimated marginal means with Bonferroni correction for multiple testing. Results were reported as odds ratios (ORs) with 95% confidence intervals (CI).<br>For each selected item, either validated in the second round or sent to the third, the percentage of respondents scoring ≥7, the IQR, and the median were calculated for each group and overall. To allow prioritization, items were classified by median into four perceived-relevance levels: Median < 7: low relevance; Median ≥ 7: moderate relevance; Median ≥ 8: high relevance; Median ≥ 9: very high relevance. Consensus was considered reached if at least 70% of responses were ≥7 and if the IQR was ≤2.5. Total consensus meant all groups met these thresholds, ; global consensus referred to the overall sample meeting them; partial consensus referred to only some expert groups meeting them.

Topic	Section	Item	Checklist Item	Location where item is reported	Citation of the text
		29	Definition and measurement of consensus		items were classified as: (a) consensual: at least 70% of participants rated an item ≥7 (≥70%≥7), and the interquartile range (IQR) was ≤ 2.5 resulting in items beeing selected and not re-rated in the third round;
		30	Information on group-specific analysis or weighting of experts (e.g., theory vs. practice, discipline-specific analysis)		For each selected item, either validated in the second round or sent to the third, the percentage of respondents scoring ≥7, the IQR, and the median were calculated for each group and overall. To allow prioritization, items were classified by median into four perceived-relevance levels: Median < 7: low relevance; Median ≥ 7: moderate relevance; Median ≥ 8: high relevance; Median ≥ 9: very high relevance. Consensus was considered reached if at least 70% of responses were ≥7 and if the IQR was ≤2.5. Total consensus meant all groups met these thresholds, ; global consensus referred to the overall sample meeting them; partial consensus referred to only some expert groups meeting them.
IV Results	Delphi process	31	Illustration of the Delphi study (e.g., in a flow chart)		The flow of participants and consensus outcomes across the three Delphi rounds is presented in Figure 1
		32	Information about special aspects during the Delphi study (e.g., deviations from the intended approach with justification)		During the Delphi study the political discussion mentioned climate change and the effects on health. It is possible that this influenced the experts' responses.
		33	Number of experts per round (both invited and participating)		Expert group Round 1 Round 2 Round 3<br>Older adults 19 13 13<br>Health & science 19 28 20<br>Urban planners 19 9 10<br>Decision-makers 16 13 13<br>Total 64 60 49
	Results	34	Presentation of the results for each Delphi round and the final results		Presented in Figures 3 4 5 6
V Discussion	Quality of findings	35	Highlighting the findings from the Delphi study		Based on these results and the literature, several recommendations can guide public policies and practices for preventing outdoor falls (Figure 7). Falls should no longer be considered only a clinical problem; they are a transversal issue of public health and urban planning. Municipalities should explicitly embed fall prevention in urban, mobility,

Topic	Section	Item	Checklist Item	Location where item is reported	Citation of the text
					and health plans with stable dedicated funding (Thurairaj et al., 2025; World Health Organization, 2007). Strong consensus highlights basic pedestrian safety dimensions as immediate, widely shared levers to reduce fall risk. Investing in systematic maintenance protocols, rapid repairs, and securing risk zones should become standard practice. Multidisciplinary actions (systematic inclusion of fall prevention in planning documents, targeted training for planners and technical agents, and monitoring tools such as observatories and participatory reporting) should also be prioritized.
		36	Validity of the results (e.g., transferability of the findings)		This study has several limitations. First, it was conducted in Normandy, France, and although the region is demographically and urbanistically representative of many European regions undergoing advanced demographic transition, priorities and perceptions may vary in other contexts, such as very dense urban areas or countries with different infrastructures and policies. Replication elsewhere is needed to test transferability. Choosing a very broad geographical area (multiple countries) could have led to a consensus emerging only on very general aspects, more widely applicable but less insightful.
		37	Reliability of the results (e.g., split half, inter-rater reliability)		Second, panel recruitment was voluntary, which may introduce selection bias toward more sensitized stakeholders. Complementary population surveys or random panels could enhance representativeness. Third, as with any Delphi, results reflect perceptions and expertise rather than objective measurement, and they have not yet been matched to field data (epidemiological, sensor-based, in situ observation).
		38	Reflection on potential limitations (e.g., number of experts, response bias)		Fourth, consensus methods inherently produce compromises. Technical details (e.g., precise surfacing specifications) or very personal issues (e.g., anxiety after a fall) may have been diluted in the final ranking even if recognized as important in the literature. In-depth qualitative interviews, exploratory walks, and in situ observations could identify latent needs or “blind spots,” refining understanding

Topic	Section	Item	Checklist Item	Location where item is reported	Citation of the text
					of still understudied dimensions (e.g., sensory overload, cognitive fatigue, combined environmental conditions).

[1] "Experts" are the participants; these can be people from academia, practice, or representatives of lived experience (e.g., patients, family members).

[2] The term "questionnaire" stands for the survey instrument regardless of whether quantitative or qualitative items are integrated or weighted.
